# Supplementary material for: Multi Evaluation of a Modified GoldNano Carb Test for Carbapenemase Detection in Clinical Isolates of Gram-Negative Bacilli
Source: Antibiotics (Basel). 2022 May 18;11(5):684. doi: 10.3390/antibiotics11050684 (PMC9137630; doi:10.3390/antibiotics11050684)
Supplement: Supplementary file 1 [file antibiotics-11-00684-s001.zip › antibiotics-1723552-supplementary.pdf]

Supplementary Materials

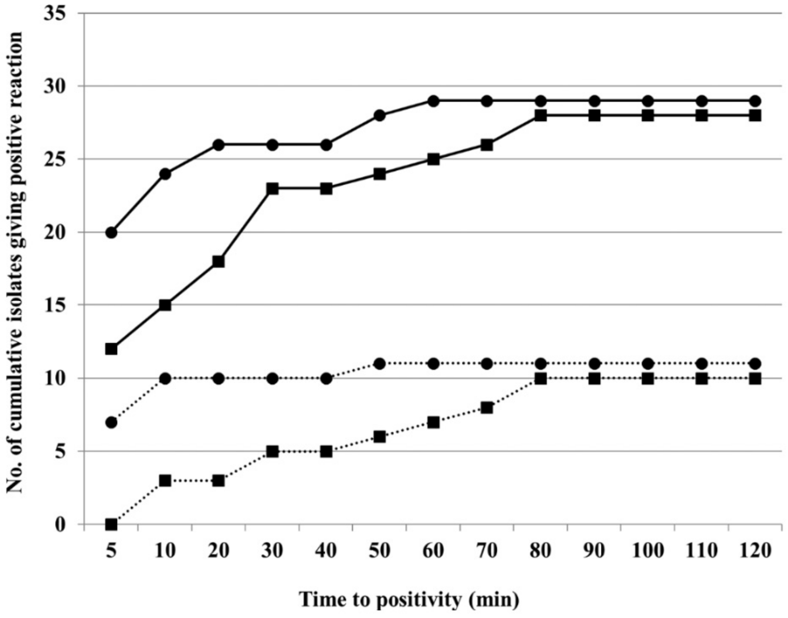

Figure S1. Time to positivity by the modified GoldNano Carb test (●) compared with the conventional GoldNano Carb test (■) among 29 carbapenemase producers (solid lines) and 11 OXA producers (dashed lines).

Table S1. Detail of Gram-negative bacilli clinical isolates collected from six hospitals

Organisms	No. of isolates from each hospital						
	**A**	**B**	**C**	**D**	**E**	**F**	**Total**
Collection period between	Jun and Jul 2019	May and Aug 2020	Feb and Aug 2019	Sep 2019 and Jan 2020	Jan and Aug 2020	Feb and Aug 2019	
Enterobacterales	105	124	94	72	53	47	495
Acinetobacter spp.	63	44	0	68	37	0	212
P. aeruginosa	59	21	0	24	21	0	125
Total	227	189	94	164	111	47	832

A & B, Northeast; C & D, Central; E, West; F, South.

Table S2. Carbapenemase genes in clinical isolates collected from the six hospitals

**Hospitals/Gram negative bacteria**	**No. of isolates with carbapenemase genes**														**Non-carbapenemases**	**Total**
	***bla*$_{OXA 48-like}$**	***bla*$_{NDM}$**	***bla*$_{VIM}$**	***bla*$_{IMP}$**	***bla*$_{OXA 23-like}$**	***bla*$_{OXA 58-like}$**	***bla*$_{IMP}$, *bla*$_{OXA 48-like}$**	***bla*$_{NDM}$, *bla*$_{IMP}$**	***bla*$_{NDM}$, *bla*$_{VIM}$**	***bla*$_{NDM}$, *bla*$_{OXA 48-like}$**	***bla*$_{NDM}$, *bla*$_{OXA 23-like}$**	***bla*$_{NDM}$, *bla*$_{OXA 58-like}$**	***bla*$_{OXA 23-like}$, *bla*$_{OXA 58-like}$**	***bla*$_{NDM}$, *bla*$_{OXA 23 like}$, *bla*$_{OXA 58 like}$**		
**A**																**227**
K. pneumoniae	14	10													22	46
E. coli	1	1													42	44
P. mirabilis															5	5
Enterobacter spp.															4	4
Klebsiella spp.															3	3
P. vulgaris															1	1
M. morganii															1	1
C. koseri															1	1
A. baumannii					27						2		1		33	63
P. aeruginosa		2	3	3				1							50	59
**B**																**189**
K. pneumoniae	19	14								1					34	68
E. coli	2	21													31	54
K. aerogenes		2														2
A. baumannii		1			32	2							3		6	44
P. aeruginosa		10	2	2											7	21
**C**																**94**
K. pneumoniae	52	8								2					8	70
E. coli	5						1			1					9	16
Proteus spp.	1														3	4
Enterobacter spp.	1														1	2
Serratia spp.															1	1
M. morganii															1	1
**D**																**164**

Hospitals/Gram negative bacteria	No. of isolates with carbapenemase genes														Non-carbapenemases	Total
	$bla_{OXA-48-like}$	bla_{NDM}	bla_{VIM}	bla_{IMP}	$bla_{OXA-23-like}$	$bla_{OXA-58-like}$	bla_{IMP}, $bla_{OXA-48-like}$	bla_{NDM}, bla_{IMP}	bla_{NDM}, bla_{VIM}	bla_{NDM}, $bla_{OXA-48-like}$	bla_{NDM}, $bla_{OXA-23-like}$	bla_{NDM}, $bla_{OXA-58-like}$	$bla_{OXA-23-like}$, $bla_{OXA-58-like}$	bla_{NDM}, $bla_{OXA-23-like}$, $bla_{OXA-58-like}$		
K. pneumoniae	5	11		1											22	39
E. coli		2													14	16
Enterobacter spp.				1											8	9
Serratia spp.															4	4
E. aerogenes															1	1
E. cloacae															1	1
S. marcescens															1	1
M. morganii															1	1
A. baumannii					51	4					1		2		9	67
A. haemolyticus		1														1
P. aeruginosa			1												23	24
**E**																**111**
K. pneumoniae	10	14													5	29
E. coli		6													10	16
Enterobacter spp.		1													3	4
C. freundii		1													1	2
Klebsiella spp.		1													1	2
A. baumannii		1			26	2						3	3	1	1	37
P. aeruginosa		5	6	7				1	1						1	21
**F**																**47**
K. pneumoniae	4	12													15	31
E. coli		8													3	11
Enterobacter spp.		4													1	5
**Total**	**114**	**136**	**12**	**14**	**136**	**8**	**1**	**2**	**1**	**4**	**3**	**3**	**9**	**1**	**388**	**832**

A & B, Northeast; C & D, Central; E, West; F, South.
